# Cotranslational Biogenesis of Membrane Proteins in Bacteria

**DOI:** 10.3389/fmolb.2022.871121

**Published:** 2022-04-29

**Authors:** Evan Mercier, Xiaolin Wang, Lena A. K. Bögeholz, Wolfgang Wintermeyer, Marina V. Rodnina

**Affiliations:** Department of Physical Biochemistry, Max Planck Institute for Multidisciplinary Sciences, Göttingen, Germany

**Keywords:** N-terminal processing, membrane targeting, membrane insertion, membrane protein topology, translocon, cotranslational folding, YidC

## Abstract

Nascent polypeptides emerging from the ribosome during translation are rapidly scanned and processed by ribosome-associated protein biogenesis factors (RPBs). RPBs cleave the N-terminal formyl and methionine groups, assist cotranslational protein folding, and sort the proteins according to their cellular destination. Ribosomes translating inner-membrane proteins are recognized and targeted to the translocon with the help of the signal recognition particle, SRP, and SRP receptor, FtsY. The growing nascent peptide is then inserted into the phospholipid bilayer at the translocon, an inner-membrane protein complex consisting of SecY, SecE, and SecG. Folding of membrane proteins requires that transmembrane helices (TMs) attain their correct topology, the soluble domains are inserted at the correct (cytoplasmic or periplasmic) side of the membrane, and – for polytopic membrane proteins – the TMs find their interaction partner TMs in the phospholipid bilayer. This review describes the recent progress in understanding how growing nascent peptides are processed and how inner-membrane proteins are targeted to the translocon and find their correct orientation at the membrane, with the focus on biophysical approaches revealing the dynamics of the process. We describe how spontaneous fluctuations of the translocon allow diffusion of TMs into the phospholipid bilayer and argue that the ribosome orchestrates cotranslational targeting not only by providing the binding platform for the RPBs or the translocon, but also by helping the nascent chains to find their correct orientation in the membrane. Finally, we present the auxiliary role of YidC as a chaperone for inner-membrane proteins. We show how biophysical approaches provide new insights into the dynamics of membrane protein biogenesis and raise new questions as to how translation modulates protein folding.

## Introduction

Membrane proteins comprise 20–30% of the cellular proteome and are critical for allowing cells to communicate with the extracellular environment. The common feature of inner-membrane proteins is the presence of transmembrane helices (TMs) which contain large patches of hydrophobic residues that are likely to misfold if not inserted into the phospholipid bilayer of the membrane. Efficient biogenesis of properly folded membrane proteins relies on multiple processes including N-terminal processing, membrane targeting, membrane insertion and folding, which are all coordinated in the cell. In bacteria like *Escherichia coli*, this coordination has to keep up with the rapid synthesis of new proteins by the ribosome, which churns out polypeptide chains at 10–20 amino acids per second on average. The rate of translation defines the time window in which a membrane protein emerging from the ribosome exit tunnel has to be recognized, processed, and targeted to the membrane to prevent misfolding. In this review, we summarize the main findings and current models of how bacteria have overcome this challenge by ensuring that all steps of membrane protein biogenesis are efficient and rapid in order to win the race against the clock as a new membrane protein is synthesized on the ribosome.

Recent insights into cotranslational protein biogenesis have revealed that protein folding starts early, when most of the nascent protein chain is still inside the ribosome (reviewed in (Bustamante et al., 2020; Cassaignau et al., 2020; Liutkute et al., 2020b)). Compaction of several nascent proteins has been observed inside the ribosome and, in some cases, the extent of folding can be quite substantial, with secondary structure elements such as α-helices and even small domains folding relatively deep inside the exit tunnel of the ribosome (Woolhead et al., 2004; Lu and Deutsch, 2005; Nilsson et al., 2015; Marino et al., 2016; Farías-Rico et al., 2018; Liutkute et al., 2020a; Agirrezabala et al., 2022). Larger structural elements can fold upon arrival at the vestibule region of the ribosome exit tunnel or outside the ribosome (Holtkamp et al., 2015; Bustamante et al., 2020; Liutkute et al., 2020b). Remarkably, an all-β protein has been shown to compact inside the exit tunnel of the ribosome as an α-helix, suggesting that the environment of the exit tunnel favors formation of α-helices (Agirrezabala et al., 2022). On the other hand, protein structures remain dynamic as long as the nascent protein is bound to the ribosome (Kaiser et al., 2011; Liutkute et al., 2020a; Agirrezabala et al., 2022). Together, these findings indicate not only a clear trend for nascent-protein folding inside the ribosome, but also a profound ability of the ribosome to alter the folding landscape of nascent proteins. How well the cotranslational folding trends elucidated for cytosolic proteins extend to membrane proteins is still unclear, but recent advances in biophysical techniques provide new insights into how nascent membrane proteins fold on the ribosome, and how the ribosome influences the different steps in membrane protein biogenesis.

**FIGURE 1:**
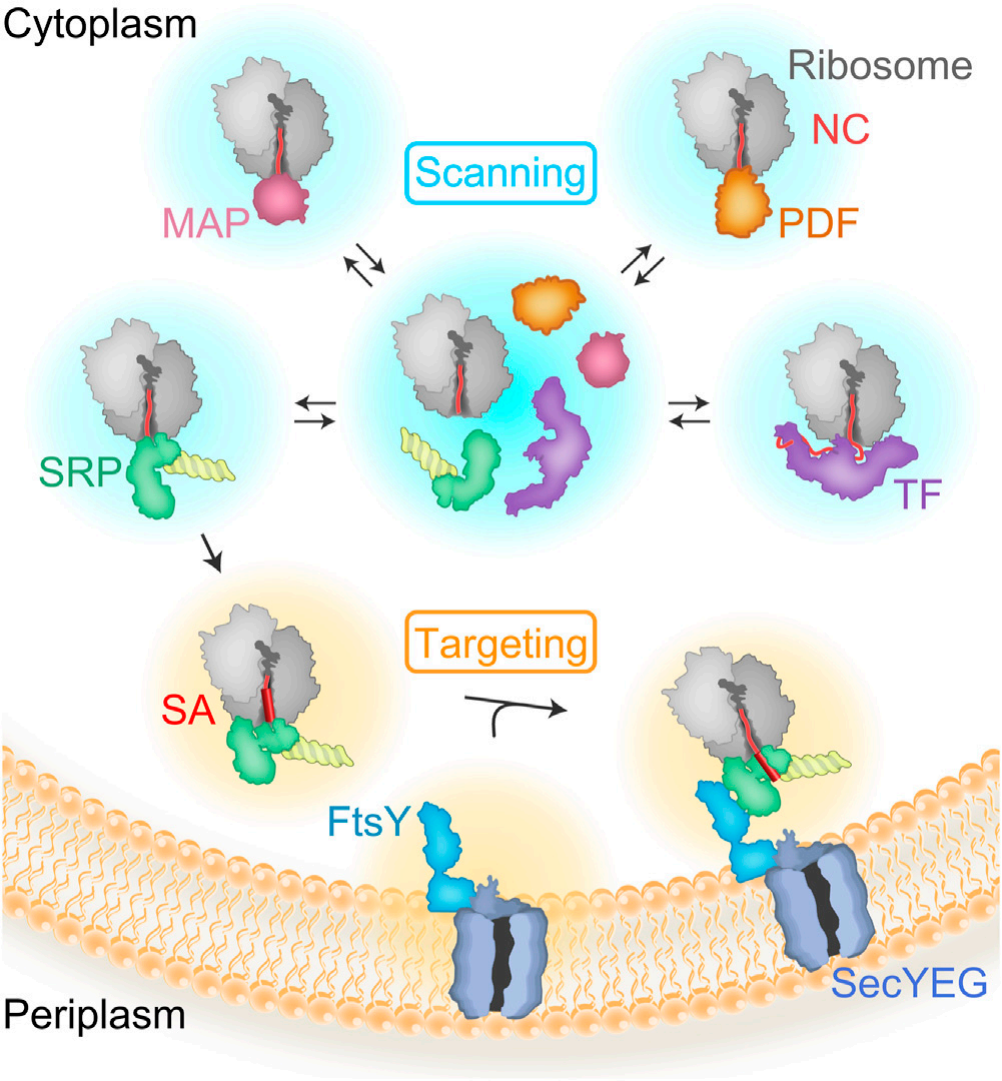
Scanning and targeting of nascent polypeptides. RBPs rapidly bind to the ribosome during ongoing translation to scan emerging nascent polypeptide chains (NC). Most of the proteins are first deformylated by PDF and then processed by MAP, which cleaves the N-terminal methionine. TF is a chaperone with preference for cytosolic or outer membrane proteins. TF and SRP compete for binding to ribosomes with short nascent chains. Nascent peptides emerging from the exit tunnel of the ribosome expose recognition motifs leading to kinetic stabilization or rejection of TF vs SRP. SRP recognizes the signal-anchor sequence (SA) and targets the RNCs to the translocon, SecYEG, with the help of the SRP receptor, FtsY.

The influence of the ribosome on cotranslational protein biogenesis does not end when the nascent protein emerges from the ribosome. Proteins called ribosome-associated protein biogenesis factors (RPBs) that facilitate nascent chain maturation and targeting bind at the tunnel exit and interact with the emerging nascent protein (Kramer et al., 2009; Giglione et al., 2015; Koubek et al., 2021). RPBs comprise peptide deformylase (PDF) and methionine aminopeptidase (MAP), which are responsible for N-terminal processing; the chaperone trigger factor (TF); and the signal recognition particle (SRP) which targets inner membrane proteins and secretory proteins to the membrane with the help of the SRP receptor (SR), FtsY (Figure 1). All RPBs scan ribosome-nascent-chain complexes (RNCs) for suitable nascent chain substrates by binding to the ribosome close to the tunnel exit where the new protein emerges (Holtkamp et al., 2012; Sandikci et al., 2013; Bornemann et al., 2014). The area near the tunnel exit, therefore, provides a platform capable of coordinating the timing and action of different RPBs. The region is also responsible for binding the SecYEG translocon, which helps nascent membrane proteins to insert into the phospholipid bilayer. The TMs of a membrane protein reach the phospholipid bilayer during ongoing translation and must attain the correct topology with respect to the membrane, and fold appropriately. We focus this review on the role of the ribosome in coordinating these different events to ensure that all processes occur in a timely manner.

## Scanning of RNCs by RPBs

### N-Terminal Processing of Nascent Cytosolic Proteins

N-terminal processing begins cotranslationally as soon as the formylated methionine at the N-terminus of a nascent chain emerges from the polypeptide exit tunnel of the ribosome (Figure 1). In the first step, the formyl group is removed by PDF, which is the prerequisite for subsequent removal of methionine by MAP. The essential protein PDF is a small metalloenzyme that carries an Fe(II) cofactor in its active site (Mazel et al., 1994; Rajagopalan et al., 1997) and contains a C-terminal helix that binds to the ribosomal protein uL22 (Chan et al., 1997; Bingel-Erlenmeyer et al., 2008; Bhakta et al., 2019; Akbar et al., 2021). Binding of PDF to the ribosome and dissociation of the complex is rapid, estimated to occur 65 times per second, which allows the enzyme to randomly scan ribosomes until it encounters an RNC carrying its reaction substrate, a formylated nascent chain (Sandikci et al., 2013; Bogeholz et al., 2021). The binding sites of PDF and MAP, which both bind at uL22, overlap, precluding simultaneous recruitment of the two proteins at their primary binding site on the ribosome (Bhakta et al., 2019). The competition between PDF and MAP enhances the scanning process by increasing the selectivity of PDF to N-formylated nascent chain (Sandikci et al., 2013). MAP interacts with the ribosome *via* positively charged amino acids, which allows MAP to evade to a secondary binding site in the presence of PDF (Sandikci et al., 2013; Bhakta et al., 2019). As the binding site of PDF lies in close proximity of the polypeptide exit tunnel, PDF can rapidly deformylate short peptide substrates starting at a length of about 50 amino acids (Giglione et al., 2015; Bogeholz et al., 2021). For stalled RNCs, however, a preferred length of deformylation is about 70 amino acids (Ranjan et al., 2017; Yang et al., 2019).

The cleavage of the formyl group is rapid, but the subsequent turnover of PDF is delayed by a slow conformational rearrangement most likely reflecting the release of the nascent chain from the active site of the enzyme (Bogeholz et al., 2021). Owing to this slow step, deformylation is rate-limiting for N-terminal processing, because MAP can only access the N-terminus after dissociation of PDF (Yang et al., 2019). Methionine is removed from about 50% of proteins in *E. coli*. There is a strong dependence on the size of the second amino acid: nascent chains with a small amino acid in this position are preferred MAP substrates (Hirel et al., 1989; Frottin et al., 2006; Xiao et al., 2010; Bienvenut et al., 2015). The retention of the nascent chain by PDF during ongoing translation on one hand and nascent-peptide folding as it moves away from the ribosome surface on the other hand, define the time window for MAP to act before it can no longer access the N-terminus (Yang et al., 2019), which may explain the incomplete methionine removal.

### Lack of Deformylation of Membrane Proteins

While proteomics studies show that most cytosolic proteins are deformylated, the only proteins that retain the formyl group to an extent greater than 50% are inner-membrane proteins (Bienvenut et al., 2015). When an inner-membrane protein is synthesized on the ribosome, it displays an SRP-specific signal sequence that is recognized by SRP as soon as the N-terminus of the nascent chain emerges from the polypeptide exit tunnel. Binding of SRP inhibits deformylation by PDF (Ranjan et al., 2017) even though PDF and SRP can bind the ribosome simultaneously (Bornemann et al., 2014). The extent of the inhibitory effect, however, seems to depend on the distance between the signal sequence and the N-terminus (Ranjan et al., 2017; Yang et al., 2019). Interference with deformylation by SRP recruitment allows bypassing of the relatively slow N-terminal processing and ensures efficient targeting of inner-membrane proteins to the membrane. After insertion into the membrane, N-termini that reach into the cytosol can potentially be deformylated posttranslationally comparable to the deformylation of model peptides (Ranjan et al., 2017).

### Competition Between RPBs at the Tunnel Exit

TF is another key player monitoring the emerging nascent chains. Interacting with short stretches enriched in basic and aromatic amino acids, TF acts as a chaperone mainly for cytosolic or outer-membrane proteins (Oh et al., 2011; Deckert et al., 2021; Koubek et al., 2021). TF consists of three domains. The N-terminal ribosome-binding domain interacts with the ribosome at protein uL23, which allows TF to arch over the polypeptide exit tunnel and accommodate nascent proteins (Kramer et al., 2002; Kristensen and Gajhede, 2003; Ferbitz et al., 2004). The peptidyl-prolyl isomerase domain facilitates cis/trans-isomeration of proline residues and the C-terminal domain contains the main chaperone activity (Kramer et al., 2002; Kristensen and Gajhede, 2003; Ferbitz et al., 2004; Merz et al., 2006). In the cell, TF is in two-to-three-fold excess over ribosomes (Lill et al., 1988) and as the K_d_ is about 0.1 µM (Raine et al., 2006; Bornemann et al., 2014), nearly all ribosomes can have one TF bound. TF dissociates rapidly from non-translating ribosomes, allowing the chaperone to scan for its substrates. On the ribosome, TF and PDF can bind at the same time due to a conformational change in TF (Bornemann et al., 2014; Bhakta et al., 2019). TF and MAP can also bind simultaneously (Bornemann et al., 2014); however, simultaneous binding of all three RPBs is only observed at very high MAP concentrations (Bhakta et al., 2019; Akbar et al., 2021). When TF encounters an RNC synthesizing one of its client proteins, TF binding on the ribosome is stabilized (Bornemann et al., 2014). Even though TF contacts the nascent chain early on (Merz et al., 2008), a stable engagement of TF starts at a nascent chain length of 100 amino acids for most proteins (Oh et al., 2011), which leaves sufficient time for N-terminal processing of proteins that are TF clients. TF and SRP can bind to the ribosome at the same time displaying partially competitive binding to the ribosome and the nascent chain (Buskiewicz et al., 2004; Eisner et al., 2006; Ullers et al., 2006; Bornemann et al., 2014). When SRP–TF–RNC complexes that carry a nascent chain displaying a signal peptide bind to the SRP receptor, FtsY, TF is displaced from the complex (Buskiewicz et al., 2004). On the other hand, TF slows down the recruitment of FtsY for suboptimal SRP substrates and reduces the affinity of SRP towards longer substrates, suggesting that if a protein has not been targeted to the membrane within a certain time, it will remain in the cytosol until it has been fully synthesized (Ariosa et al., 2015). Thus, interplay between TF and SRP leads to efficient selection of nascent chains for different pathways.

## Biogenesis of Inner-Membrane Proteins

### Membrane Targeting

Bacterial proteins which are destined for the inner membrane, the outer membrane, or the periplasmic space are first targeted to the inner membrane through one of three pathways. The Tat and SecA/SecB pathways help to translocate proteins across the inner membrane and act largely after synthesis of the client protein is completed (reviewed in (Frain et al., 2019)). The SecA/B pathway exports proteins in an unfolded state and these clients then go on to fold in the periplasm or insert into the bacterial outer membrane (reviewed in (Smets et al., 2019)). The Tat pathway exports folded proteins from the cytosol and tends to handle proteins which either fold rapidly in the cytosol or bind cytosolic cofactors (reviewed in (Kudva et al., 2013)). Inner-membrane proteins, on the other hand, are targeted by the SRP pathway that guides actively translating ribosomes to the Sec translocon (SecYEG in bacteria) with the assistance of FtsY.

Targeting through any of these pathways requires a hydrophobic signal sequence located near the N-terminus. Signal sequences for the Tat and SecA/B pathways include positively charged amino acids N-terminal to the hydrophobic segment, which is included in the SRRxFLK consensus sequence for Tat substrates (Kudva et al., 2013; Palmer and Stansfeld, 2020). For these posttranslational export pathways, hydrophobic signal sequences are inserted into the membrane and cleaved at a peptidase recognition motif located downstream of the hydrophobic segment. Signal peptide cleavage disconnects the exported protein from the inner membrane and allows it to find its proper place in the periplasm, outer membrane, or beyond. Inner-membrane proteins are targeted by the SRP pathway (reviewed in (Kuhn et al., 2017; Steinberg et al., 2018); signal sequences of the SRP pathway do not all contain positively charged amino acids at the N-terminus, and they tend to be more hydrophobic than those of the other export pathways (Cristóbal et al., 1999; Kudva et al., 2013). The SRP-specific signal sequence is not a conserved sequence motif, but a hydrophobic segment with high propensity to form an α-helix (Lee and Bernstein, 2001; Adams et al., 2002; Peterson et al., 2010; Robinson et al., 2012). The first transmembrane domain (TM1) of an integral membrane protein is typically used for membrane targeting and is referred to as a signal-anchor sequence (SA). Some membrane proteins are targeted by a signal sequence that is later cleaved off by leader peptidase; for these proteins, the second hydrophobic segment will become the first TM of the mature protein (e.g. CyoA (Celebi et al., 2008), FliP (Pradel et al., 2004)). During targeting, SRP interacts with the signal sequence and protects it from the cellular environment, thus helping to prevent aggregation or misfolding of the hydrophobic segment.

**FIGURE 2:**
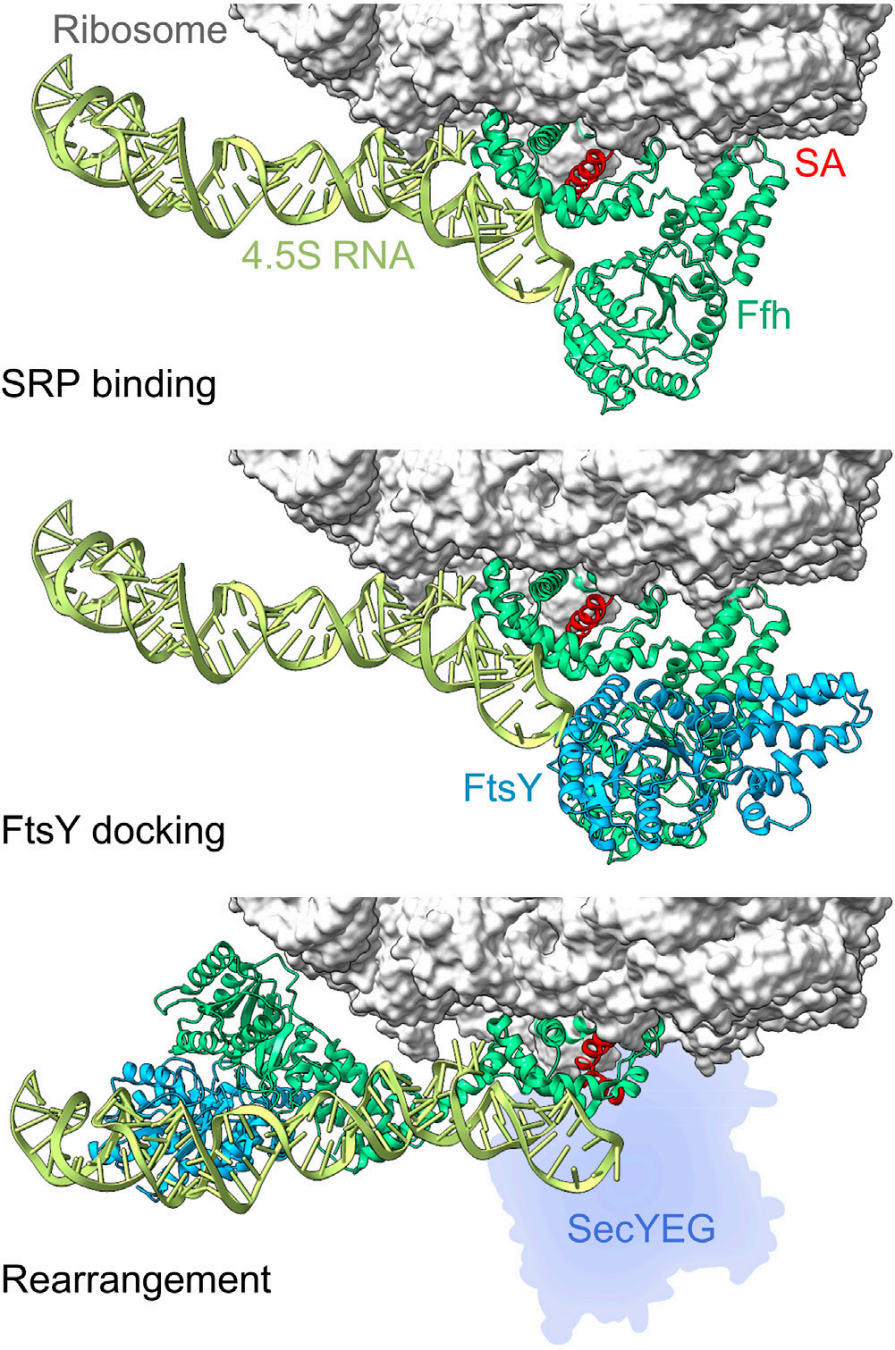
Conformational rearrangements of SRP and FtsY on the ribosome. Top panel: cryo-EM structure of the SRP binding complex at the ribosome with the M-domain of Ffh (green) recognizing the signal-anchor sequence (SA, red) emerging from the ribosome (gray). Ffh NG domain contacts the proximal end of the 4.5S RNA while the M-domain interacts with ribosomal proteins uL23 and uL29 as well as the 23S rRNA (PDB 5GAF (Jomaa et al., 2016)). Middle panel: Initial docking of FtsY on the RNC-SRP complex involves interactions between the NG domains of FtsY (blue) and Ffh (green) (PDB 5GAD (Jomaa et al., 2016)). Bottom panel, NG domains of Ffh and FtsY relocate towards the distal end of the 4.5S RNA (PDB 5NCO (Jomaa et al., 2017)), promoting handover of the RNC to the translocon (silhouette; not in the structure, inferred from PDB 5GAE (Jomaa et al., 2016)).

SRP is an RNA-protein complex which, in *E. coli*, comprises 4.5S RNA and a protein, Ffh. The 4.5S RNA is 114 nucleotides long and folds into a stem-loop structure, while the Ffh protein contains an NG domain which binds and hydrolyzes GTP, a methionine-rich M-domain, and a flexible linker connecting the two larger domains (Schaffitzel et al., 2006). Ffh binds near the proximal end of 4.5S RNA and the resulting SRP complex binds to the ribosome at the peptide tunnel exit. The M-domain contacts ribosomal proteins uL23 and uL29, as well as 23S rRNA in the vicinity of the exit tunnel (Figure 2). The flexible C-terminus of Ffh protrudes into the interior of the exit tunnel where it contacts uL23 (Jomaa et al., 2016; Denks et al., 2017). The affinity (K_d_) of SRP to ribosomes that do not carry a nascent chain is about 100 nM, (Bornemann et al., 2008; Zhang et al., 2010)). In a translating ribosome, the growing nascent chain of about 25 aa length displaces Ffh from its uL23 contact inside the exit tunnel (Denks et al., 2017), but SRP remains bound to the ribosome in a stand-by state at the exit tunnel. This enables SRP to sense a nascent protein in the exit tunnel of the ribosome, and facilitates binding of the signal sequence to the M domain of SRP immediately upon emergence from the ribosome (Halic et al., 2006; Schaffitzel et al., 2006; Hainzl et al., 2011). Recognition of the signal sequence results in a high-affinity complex whose thermodynamic stability is somewhat sensitive to the hydrophobicity of the signal sequence (K_d_ = 1–10 nM, (Bornemann et al., 2008; Zhang et al., 2010; Mercier et al., 2017)). The high thermodynamic stability of SRP-ribosome and SRP-RNC complexes (K_d_ = 1–100 nM), along with the large cellular concentration of ribosomes in *E. coli* (20–45 µM (Rudorf and Lipowsky, 2015)), indicate that all SRP complexes in the cell are bound to translating ribosomes.

Because the concentration of SRP is about 400 nM in *E. coli* (Kudva et al., 2013), SRP can occupy only 1–2% of all ribosomes at any time, and this generates a potential predicament for SRP which has to bind hydrophobic signal sequences as they emerge from the ribosome. The solution to the problem lies in a rapid scanning function of SRP that enables it to bind and dissociate from ribosomes 5–10 times per second (Holtkamp et al., 2012; Mercier et al., 2017). Detection of a non-signal sequence-containing peptide causes SRP to reject the RNC as a substrate, while a hydrophobic signal sequence induces a switch in SRP from scanning mode to targeting mode (Holtkamp et al., 2012). The switch has been observed for actively translating ribosomes, and occurs after the ribosome has incorporated 40–50 amino acids, and likely depends on where the signal sequence is located relative to the N-terminus (Noriega et al., 2014; Schibich et al., 2016; Mercier et al., 2017). In targeting mode, the SRP-RNC complex is kinetically stabilized (k_off_ = 0.03–0.1 s^−1^), which ensures that SRP remains bound until the RNC has been targeted to the membrane, and effectively protects the hydrophobic signal sequence (Holtkamp et al., 2012; Noriega et al., 2014; Saraogi et al., 2014; Mercier et al., 2017).

The SRP-RNC targeting complex is then directed to the membrane where FtsY is bound at the SecYEG translocon. FtsY contains an intrinsically disordered A domain at the N-terminus that interacts with phospholipids and SecYEG, as well as an NG domain homologous to that in SRP protein Ffh, which enables binding of FtsY to SRP (Figure 2) (Stjepanovic et al., 2011; Lakomek et al., 2016). Activation of FtsY is enhanced by anionic phospholipids (phosphatidylglycerol and cardiolipin), and binding of the A domain to the membrane-embedded translocon induces a conformational rearrangement of FtsY and effectively tethers the SRP-receptor to the translocon (de Leeuw et al., 2000; Lam et al., 2010; Stjepanovic et al., 2011; Draycheva et al., 2016). The A domain remains intrinsically disordered, thus providing a volume around the translocon where the NG domain can search for SRP-bound targeting complexes (Draycheva et al., 2016; Lakomek et al., 2016). Binding of the SRP-RNC targeting complex to the FtsY-translocon complex is driven by interaction of the NG domains in SRP and FtsY. In the complex, the NG domains of Ffh and FtsY relocate together towards the distal end of the 4.5S RNA and vacate a space in the vicinity of the tunnel exit for SecYEG (Figure 2C). This weakens SRP-RNC binding to Ffh-SecYEG, promoting handover of the RNC to the translocon (Jomaa et al., 2016; Jomaa et al., 2017; Draycheva et al., 2018). The details of handover of the nascent chain from SRP to SecYEG are unclear, but it seems to require GTP-bound SRP and FtsY, while GTP hydrolysis occurs after handover of the nascent chain in order to recycle SRP and FtsY for another round of targeting (Valent et al., 1998; Zhang et al., 2010).

### Membrane Insertion

Insertion of TMs into the phospholipid bilayer takes place at the translocon. The core translocon is a ternary transmembrane protein complex composed of proteins SecY, SecE and SecG in bacteria (Denks et al., 2014). The largest component, SecY, is comprised of 10 α-helices that form a channel across the inner membrane (Van den Berg et al., 2004). The proteins SecE and SecG are both small, each comprising two α-helices, and are located at the periphery of SecY (Park et al., 2014; Tanaka et al., 2015). The translocon can open in two ways. For protein export across the inner membrane, the small plug domain in SecY that docks onto the hydrophobic ring in the middle of the pore can be displaced into periplasm (Zimmer et al., 2008; Park et al., 2014). To permit insertion of TMs into the phospholipid bilayer, the two halves of the translocon can move apart, opening the lateral gate formed by TM2 and TM7 and exposing the pore of SecY to the phospholipid bilayer (Egea and Stroud, 2010; Frauenfeld et al., 2011; Ge et al., 2014).

Upon targeting, the translocon docks at the tunnel exit of the ribosome, with its cytoplasmic loops occupying space in the vestibule of the ribosomal exit tunnel (Frauenfeld et al., 2011; Jomaa et al., 2016). In this complex, the peptide exit tunnel forms a continuous conduit with the translocon pore that allows nascent proteins to pass directly from the ribosome to the translocon. Inside this conduit, TMs can fold as soon as they pass the constriction formed by ribosomal proteins uL4 and uL22, about 25 Å away from the peptidyl transferase center (PTC) (reviewed in (Liutkute et al., 2020b)). This is supported by compaction of nascent membrane proteins observed in eukaryotic and prokaryotic ribosomes through distance-dependent FRET measurements and crosslinking to proteins in the exit tunnel (Woolhead et al., 2004; Robinson et al., 2012). The conduit formed by the ribosome-translocon complex allows TMs to pass directly from the peptide exit tunnel of the ribosome into the translocon pore. This permits a nascent membrane protein to span a distance of about 140 Å from the PTC to the periplasmic side of the translocon in an environment largely shielded from the aqueous environment of the cell. Because the translocon binds to the ribosome partially in the vestibule, the space available for folding a membrane protein in the ribosome is restricted compared to a cytosolic domain. Experiments with eukaryotic microsomes show that nascent membrane proteins remain in a compacted state upon entering the translocon, indicating that TMs insert as helices into the translocon pore (Woolhead et al., 2004). The dynamic properties of TMs inside the ribosome have not been characterized in detail, and it is possible that they exhibit breathing-type motions between compact α-helical and extended conformations (Liutkute et al., 2020b).

### Membrane Protein Topology

**FIGURE 3:**
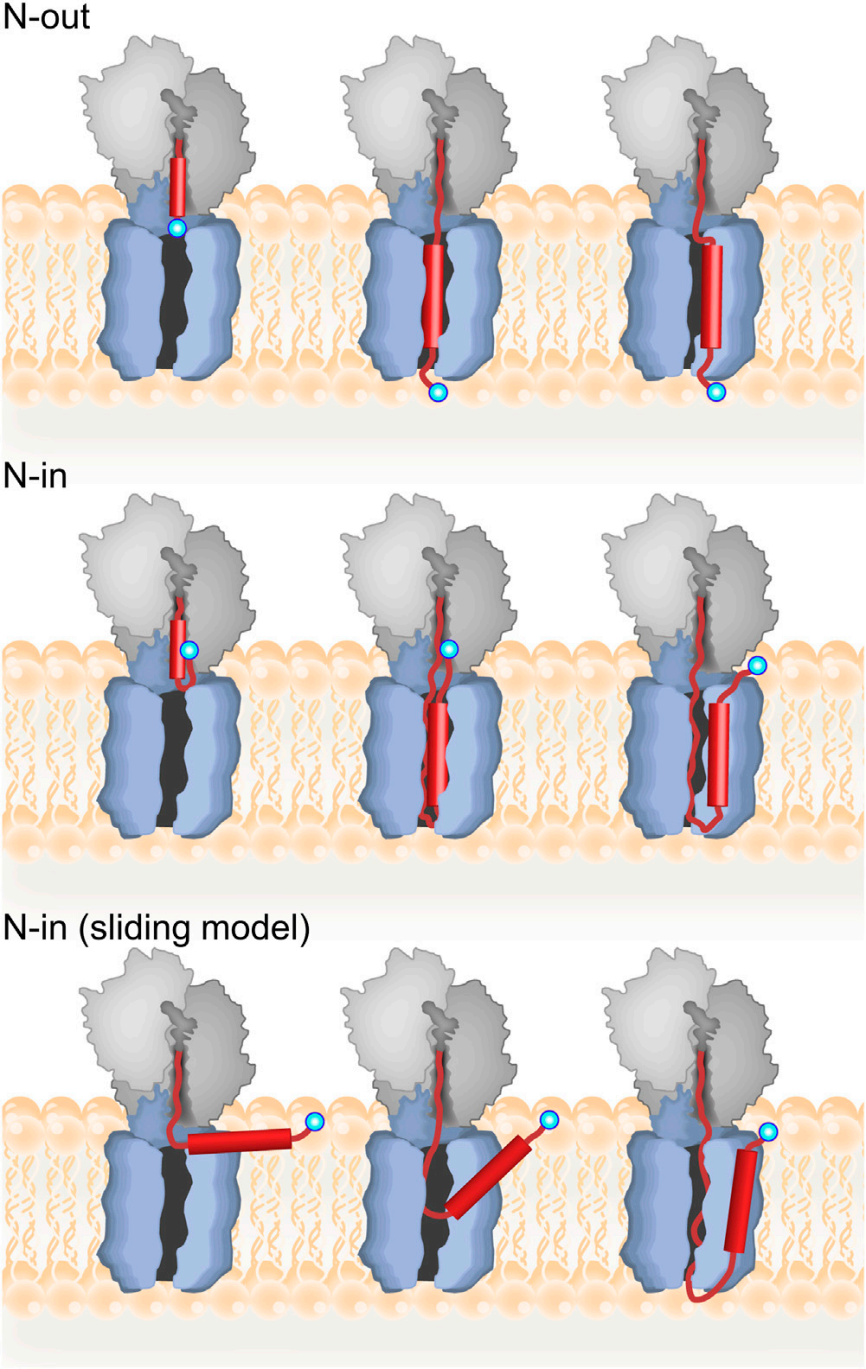
Topology of inner-membrane proteins. Top panel: model for cotranslational N-out TM insertion where the TM inserts first into the translocon prior to membrane integration. The nascent protein is depicted in red with the TM as a cylinder, and the N-terminus indicated by a blue circle. Middle panel: model for N-in TM insertion with inversion occurring in the ribosome prior to translocon insertion and then membrane integration. Bottom panel: alternative “sliding model” for N-in insertion where the TM does not insert into the translocon. For further discussion, *see* text.

TMs can insert into the membrane in two basic topologies: with the N-terminus pointing towards the cytosol (N-in) or towards the periplasm (N-out) (Figure 3). The principal determinant for TM topology is the location of positively charged amino acids within the TM, with regions of net positive charge retained on the cytosolic side of the membrane, known as the positive-inside rule (von Heijne, 1989; 1992). Because cotranslational deformylation of membrane proteins is inhibited by SRP during targeting, the N-terminus does not carry a charge and should not contribute to topology during insertion (Ranjan et al., 2017). Several factors influencing the efficiency of the positive-inside rule have been discussed, including strength of the positive charge, hydrophobicity of the TM, membrane potential, and phospholipid composition of the membrane (reviewed in (Higy et al., 2004; Kuhn et al., 2017; Spiess et al., 2019)). The simplest case is insertion with N-out topology, because no inversion of the TM is required. In this case, the loop or region preceding the TM is negatively charged or neutral, while the loop following the TM, which will be retained on the inside of the inner membrane, has a net positive charge. Retention of positive charges is favored by electrostatic interaction with negatively charged phospholipids in the inner membrane (Bogdanov et al., 2009; Dowhan et al., 2019), as well as an electric potential across the inner membrane (Cao et al., 1995), and, at least in eukaryotes, amino acids in the translocon (Junne et al., 2007).

The exact mechanism of TM insertion into the phospholipid bilayer is not clear (Cymer et al., 2015). For N-out topology, one can imagine a TM transitioning seamlessly from the ribosome into the translocon in a head-on fashion, all while the protein is being synthesized on the ribosome. This process has been monitored directly by real-time fluorescence-based measurements with high-efficiency *in-vitro* translation (Mercier et al., 2020). These experiments used distance-dependent FRET measurements to show that a FRET acceptor linked to the N-terminus of the N-out membrane protein LepB approaches a FRET donor positioned on the cytoplasmic side of the translocon, followed by insertion of TM1 in an N-out orientation, which causes a FRET decrease. Analysis of these results revealed that the simplistic description above is largely correct. In particular, kinetic analysis of these experiments indicated that translocon insertion of nascent membrane proteins is rate-limited by translation. Thus, no kinetic barrier restricts transition of the TM from the ribosome to the translocon pore. Interestingly, detailed analysis of translation rates revealed a strong pause in mRNA translation just prior to translocon insertion of the first TM. The cause of this pause is unclear, but it appears to be encoded in the mRNA since translation was carried out in an *in-vitro* system lacking any regulatory factors (Mercier et al., 2020).

Insertion of a TM with N-in topology is more complex as it requires TM inversion. In principle, inversion could result either from retention of the N-terminus on the inside of the membrane or could occur after initial insertion in an N-out orientation (Figure 3). Real-time FRET and protease protection experiments suggest that for the N-in membrane protein EmrD, the FRET acceptor linked to the N-terminus approached the FRET donor on the cytoplasmic side of the translocon to yield a high-FRET state, and then remained in this state throughout cotranslational insertion of TM1 with N-out topology (Mercier et al., 2020). Thus, insertion of TM1 with N-in topology followed retention, rather than insertion/inversion, pathway (Figure 3). That study also showed that inversion of the N-terminus occurs early, before the N-terminus of EmrD leaves the peptide exit tunnel of the ribosome. Moreover, very little FRET is observed between the cytoplasmic side of the translocon and the N-terminus of EmrD when it reaches 50 aa length, indicating a compact nascent chain that tends towards the N-in topology already at this length inside the exit tunnel of the ribosome.

The precise conformation of the inverted N-terminus inside the ribosomal exit tunnel is unclear. Since formation of the first α-helix should be possible early in the exit tunnel (*see* above), the simplest inverted conformation would be a looped structure with the N-terminus pointing in the direction of the PTC in the ribosome. Stabilization of the positively charged N-terminus could be accomplished by electrostatic interactions with negatively charged rRNA in the exit tunnel. This would suggest that the ribosome could also participate in establishing the positive-inside orientation and actively contribute to membrane protein topology before nascent membrane proteins emerge from the ribosome. Consistent with this idea, coarse-grained molecular dynamics simulations have revealed that electrostatic repulsion of negatively charged amino acids from the ribosome helps to drive cotranslational translocation of nascent proteins across the membrane (Niesen et al., 2018). Interestingly, cotranslational experiments revealed that during N-in insertion of EmrD, the ribosome paused during translation at or near codon 48 of the mRNA, at a length where the nascent chain is most likely attaining the inverted conformation (Mercier et al., 2020). Thus, the pause in mRNA translation, which is encoded in the mRNA and independent of the SecYEG insertion machinery, may provide additional time to stabilize the inverted N-in conformation of EmrD. It is not clear if formation of more complex inverted conformations (e.g. helix-turn-helix motifs) are possible in the exit tunnel, since the width of the exit tunnel is in the range of 10–13 Å, while two α-helices would require 15–20 Å depending on the side-chain conformers (O'Shea et al., 1991). While the ribosome exit tunnel widens significantly at the vestibule (up to 18 Å), binding of SecY in the vestibule constricts this space and the ribosome-translocon conduit remains narrow throughout (5–10 Å) (Frauenfeld et al., 2011). Thus, insertion with N-in topology would involve bending of the TM within the conduit of the ribosome-translocon complex or, conversely, involve lateral gate opening in SecY to provide the necessary space for acrobatics of a rigid TM.

Findings from real-time FRET measurements are in contrast to endpoint observations from ER insertion assays in COS-1 cells, which indicate that inversion occurs after initial N-out insertion of TM1 (Goder and Spiess, 2003). These studies take advantage of the glycosylation machinery present in the ER, which can modify specific sites in a region following the TM only if the TM is inserted into the ER membrane with N-in topology. In these experiments, inversion to an N-in topology is favored by increasing the time required for protein synthesis, either by elongating the C-terminal region after the TM, or by addition of cycloheximide, a reversible inhibitor of ribosomal protein synthesis. In addition, more hydrophobic TMs invert with lower efficiency than TMs with lower hydrophobicity. These findings point towards a mechanism where the TM first inserts in N-out configuration and inversion occurs later. This mechanism is also supported by chemical probing, crosslinking, and fluorescence quenching experiments performed with stalled RNCs on mammalian ER microsomes (Devaraneni et al., 2011). Here, exposure of the nascent-chain N-terminus on the luminal side of the membrane was observed at intermediate nascent-chain lengths, but at longer lengths luminal exposure was reduced. These results advocate for N-out insertion of the TM prior to inversion.

The two scenarios, where inversion can occur either by looping of the N-terminus while still inside the ribosome, or N-out insertion of the TM followed by flipping, are reflected in a kinetic model for TM inversion based on coarse-grained molecular dynamics simulations (Zhang and Miller, 2012). The results from these simulations indicate that both flipping mechanisms are possible, with the looping mechanism preferred at faster translation rates, while reducing the simulated rate of nascent chain elongation increased the fraction of trajectories showing N-out insertion and subsequent TM inversion. This kinetic description helps to reconcile the findings from real-time TM insertion experiments and endpoint assays by revealing that the insertion/inversion mechanism would be favored if *in-vitro* translation was slow, or if inversion was studied using stalled RNCs. This is also evident in real-time measurements, where an N-out intermediate was found to form posttranslationally when translation of an N-in protein was terminated after the incorporation of 85 amino acids (Mercier et al., 2020). While this intermediate could be identified as off-pathway through kinetic analysis of real-time data, this is not possible for endpoint assays. It is also noteworthy that the real-time FRET studies were performed using a translation/insertion system from *E. coli*, while the other studies have employed eukaryotic ribosomes, which have a somewhat different exit tunnel anatomy. The part of the peptide exit tunnel close to the vestibule is more narrow in eukaryotic than in bacterial ribosomes, so there would be less space available for looping of the nascent chain N-terminus inside the ribosome of eukaryotes (Dao Duc et al., 2019). In addition, real-time measurements were made with nascent polytopic membrane proteins, while the other studies have used exported proteins with a single TM: a model hydrophobic sequence rather than a naturally occurring amino acid sequence (Goder and Spiess, 2003; Devaraneni et al., 2011; Zhang and Miller, 2012). For exported proteins, TM inversion may be related to translocation of the protein C-terminus across the membrane, which is generally a posttranslational process, and thus it is unclear how strictly these findings should be applied for integral membrane proteins which are inserted cotranslationally.

In addition to cotranslational TM inversion, posttranslational inversion may play an important role in determining the membrane-protein topology of some integral membrane proteins. For example, the dual-topology protein EmrE has been suggested to invert after membrane insertion (Woodall et al., 2017). Examination of EmrE by *in-vivo* chemical probing, however, revealed that protomer topology is unaffected by the topology of the dimerization partner, indicating that protein topology is determined prior to dimerization and complete topological inversion is unlikely *in vivo* (Fluman et al., 2017). Interestingly, addition of a single positive charge at the C-terminal TM5 of EmrE is capable of inverting the final topology of the protein, suggesting that inversion may occur very late during protein synthesis, probably after insertion of several TMs (Seppälä et al., 2010). *In-vitro* measurements with proteoliposomes suggested that inversion of the membrane protein LacY occurs posttranslationally, and can be induced either by changing the lipid composition or by LacY phosphorylation (Vitrac et al., 2013; Vitrac et al., 2017). In addition, an internal TM (TM3) of human aquaporin was shown to insert with different topology if the protein was truncated after 3 TMs, or if 4, 5 or 6 TMs were translated (in *Xenopus* oocytes), indicating that TM topology can be influenced by elements that are translated later (Lu et al., 2000). Further work should clarify how common posttranslational TM inversion is, and whether it occurs shortly after TM insertion or awaits specific cellular signals.

### TM and Translocon Dynamics

In order for a TM to transit from the pore of the translocon into the phospholipid bilayer it must pass through the lateral gate. Opening of the lateral gate has been observed upon insertion of a TM into the translocon using fluorescence quenching, indicating that lateral gate opening is induced during cotranslational insertion, likely by the TM itself (Ge et al., 2014). RNCs carrying nascent membrane proteins have been visualized in complex with translocons using cryo-EM, and demonstrate that the lateral gate can be opened to different degrees during TM insertion (Frauenfeld et al., 2011; Bischoff et al., 2014; Park et al., 2014; Kater et al., 2019). The modest resolution of these structures (6–10 Å) makes it difficult to place the substrate TMs unequivocally, but they appear to occupy positions at the lateral gate, partially in the lateral gate and partially in the phospholipid bilayer, or in the lipid bilayer at the outside of the lateral gate. Thus, the information available from cryo-EM indicates that both the lateral gate and substrate TM are dynamic during the process of membrane integration. Two groups have carried out single-molecule FRET studies to characterize lateral-gate conformations, and these studies suggest a very dynamic picture of the lateral gate (Fessl et al., 2018; Mercier et al., 2021). Specifically, in addition to open and closed conformations, at least two partly open conformations are evident, and both studies confirm that lateral gate opening occurs even in the absence of substrates. Kinetic analysis of conformational changes at the lateral gate indicated that spontaneous lateral gate opening is rapid, occurring multiple times per second (Mercier et al., 2021). This is fast enough to support cotranslational insertion of TMs, each one requiring about 2 s for synthesis by the ribosome. Thus, acceleration of lateral gate opening by the ribosome or TM is not necessary for efficient phospholipid insertion. Rather, stochastic lateral gate opening permits TMs to integrate into the phospholipid bilayer when the nascent chain has reached a sufficient length (Mercier et al., 2021). Thus, the timing of TM integration into the membrane is dictated by the ribosome and the speed at which it translates the mRNA.

An alternative model for TM insertion proposes that TMs do not enter the pore of the translocon (Cymer et al., 2015). In this model, TMs exit the ribosome and adsorb at the membrane interface, parallel to the membrane surface, consistent with the notion that TMs tend to adsorb to the membrane interface (Ulmschneider et al., 2014). Only periplasmic loops enter the translocon pore where they are translocated across the membrane as the TM integrates into the phospholipid bilayer and orients itself perpendicular to the plane of the membrane. This “sliding model” is which TMs slide along the outside of the lateral gate can be distinguished from the “translocon-insertion” model based on the length of nascent chain required for N-out versus N-in insertion. For the translocon-insertion model, N-out TM insertion should occur at shorter nascent chain lengths than N-in, because N-out insertion requires the nascent protein to traverse the membrane only once (Figure 3). For the sliding model, however, both N-out and N-in insertion should occur at similar nascent-chain lengths. Support for the sliding model is found in detailed force-profile analysis (FPA) which indicates that TMs stop generating pulling force when they are about 45 amino acids away from the PTC, regardless of orientation (N-in vs N-out; (Nicolaus et al., 2021)). This nascent chain length would mean interaction with lipids rather than TM insertion in its correct topology. However, other FPA experiments clearly demonstrate a biphasic force profile generated by TM insertion, with the first peak attributed to translocon insertion, and the second (larger) peak to membrane insertion, thus supporting the canonical translocon-insertion model (Ismail et al., 2012; Niesen et al., 2018). As mentioned above, the translocon-insertion model is also supported by real-time measurements of cotranslational insertion, which indicate that insertion monitored *via* FRET, and protection against protease digestion occur faster for TMs with N-out topology than N-in (Mercier et al., 2020).

It is noteworthy that the two models are not mutually exclusive. A TM could maintain continuous access to the lipid bilayer (Nicolaus et al., 2021) – as suggested by the sliding model – if rapid lateral gate opening allowed the TM to fluctuate between positions in the translocon pore and the phospholipid bilayer, thus satisfying both models. This idea would be consistent with the weak density for nascent-chain TMs near the lateral gate observed in cryo-EM (Kater et al., 2019). With the demonstration of rapid lateral gate opening comes the possibility that TMs may fluctuate between the translocon pore and the phospholipid bilayer during insertion (Mercier et al., 2021). The ability of TMs to rapidly sample multiple positions in the lipid bilayer and the translocon could have a significant impact on the cotranslational folding of polytopic membrane proteins by allowing the TMs to sample different positions in an incomplete helix bundle, before finding the correct position. Understanding potential fluctuations of the TM between the translocon pore and the phospholipid bilayer and impacts on folding will require future single-molecule measurements focusing on TM behavior.

### Cotranslational Folding of Membrane Proteins

Insights into how polytopic membrane proteins start to fold became available recently through the FPA technique (Nicolaus et al., 2021). In general, elements of polytopic membrane proteins are inserted or translocated when they are about 45 amino acids away from the PTC (Mercier et al., 2020; Nicolaus et al., 2021). In some cases, however, TM insertion can be delayed (in terms of amino acid length) by positively charged amino acids that are likely retained by interactions with negative charges on the ribosome and/or phospholipids (Nicolaus et al., 2021). An important finding is that mutations at key positions in TMs decrease the propensity of the following TM to insert into the phospholipids, suggesting that TMs are stabilized in the phospholipid bilayer by TM-TM interactions (Cymer and von Heijne, 2013; Nicolaus et al., 2021). Based on the nature of the technique, these pulling forces are detected in partially synthesized proteins and the TM-TM interactions identified are, therefore, formed before the protein can be completely folded. The importance of these interactions is supported by single-molecule atomic-force microscopy (AFM) experiments, where insertion of LacY in membranes containing SecYEG follows a preferred mechanism where a single C-terminial helix inserts first, followed by neighbouring helices (Laskowski et al., 2021). Another single-molecule force spectroscopy study utilized magnetic tweezers to monitor transmembrane protein folding in bicelles, albeit in the absence of translocon (Choi et al., 2019). The results of this work suggest that folding of *E. coli* GlpG as well as the human ß_2_-adrenergic receptor proceed from the protein N-terminus to the C-terminus. A variety of pathways may, therefore, be accessible to different transmembrane proteins. The TM-TM interactions can be transient during ongoing translation. By analogy to cotranslational protein folding of cytosolic proteins, nascent TMs may remain in a dynamic state, where TM-TM interactions rearrange rapidly until enough elements have reached the correct location and stable interactions are assembled. Maintaining a nascent membrane protein in a quasi-stable structure would facilitate formation of rather complicated folds where for example, a TM interacts with TMs that do not immediately precede it, or TMs are intertwined rather than parallel. While the compaction of TMs into the their native fold in the membrane remains an open question, this TM-centered view of folding says nothing of the loops, which may remain dynamic to facilitate interactions with ligands and/or other proteins. Conversely, some loops may actually contribute to stabilizing the native folded structure of membrane proteins, perhaps being buried within other loops (Tastan et al., 2009).

While the application of FPA has given us detailed insights into critical events during polytopic membrane protein folding, it does not provide kinetic information about the folding process, as FPA relies on intermediates generated by programmed ribosome stalling. Determining the speed of TM insertion, loop translocation, and TM-TM interactions is important to understand the mechanism of folding in the membrane, and which steps occur co- vs. posttranslationally. This becomes even more important when we consider the influence of ongoing translation, where TMs and loops reach their respective locations only after they have been synthesized and traversed the peptide exit tunnel and (potentially) the translocon. Extrapolating the trend observed for real-time insertion of TM1 would suggest that translation on the ribosome is rate-limiting for all other steps in protein folding. This idea is supported by the timescales of loop motions and larger domain motions which are generally on the microsecond-millisecond timescales (Henzler-Wildman and Kern, 2007), much faster than the relatively low rate of protein synthesis. Thus, the ribosome will dictate when each new TM can join the nascent protein as it folds in the phospholipid bilayer, and folding events that may require significant time, such as formation of knots or interweaving of TMs, may be modulated by encoded regions in the mRNA that are translated slowly.

Another interesting question is where membrane protein folding occurs. Some loops are long enough to permit TMs to move away from the translocon and equilibrate in the lipid bilayer. Other loops, however, are so short that a preceding TM would have to wait at the lateral gate before both TMs can insert together. Cryo-EM structures indicates that a nascent membrane protein could fold in the vicinity of the translocon, perhaps even using TMs of SecY to chaperone folding (Frauenfeld et al., 2011; Bischoff et al., 2014; Park et al., 2014; Kater et al., 2019). Folding in the vicinity of the translocon would benefit proteins with internal soluble domains, where N-terminal TMs would have to wait for later insertion of C-terminal TMs, and the translocon would act as a chaperone to hold early TMs until arrival of late TMs. The TMs that follow the soluble domain may require an additional round of targeting by SRP, which has been observed in some membrane proteins (Schibich et al., 2016).

### Membrane Protein Chaperone YidC

**FIGURE 4:**
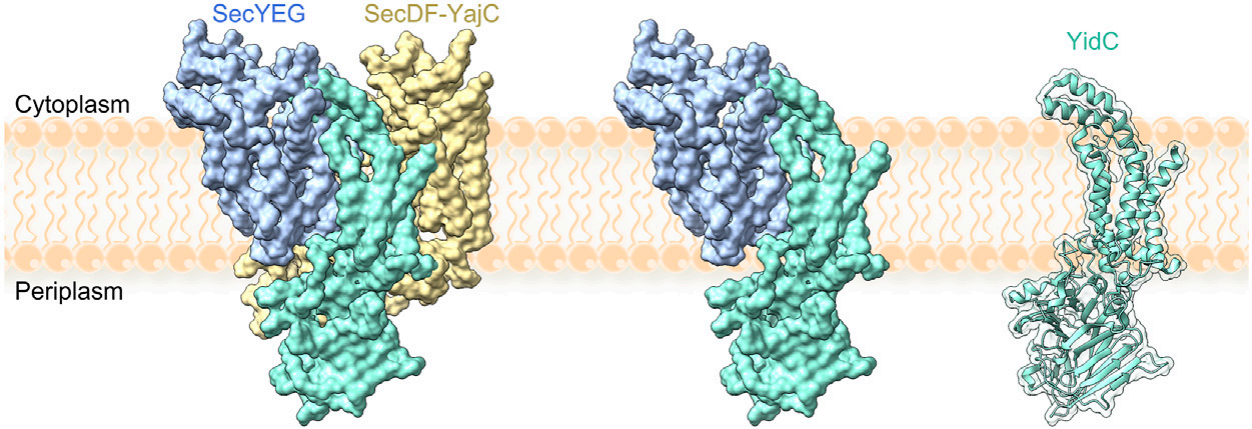
YidC in complex with the holotranslocon SecYEG–SecDF–YajC (left), with SecYEG (middle) and alone (right). Models were constructed by alignment of the holotranslocon (PDB: 5MG3 (Botte et al., 2016)) with a recent structure of YidC (PDB: 6AL2 (Tanaka et al., 2018)). TM1 of YidC is missing from all structures.

Cotranslational folding of membrane proteins at the translocon may be facilitated by the membrane-protein chaperone YidC. YidC is an essential protein in bacteria (Gerdes et al., 2003), with homologues in mitochondria (Oxa1), chloroplasts (Alb3), ER membrane (GET1, EMC3 and TMCO1) and archaea (Ylp1 or DUC160) (Borowska et al., 2015; Anghel et al., 2017; Kuhn and Kiefer, 2017; McDowell et al., 2021). YidC is comprised of 6 TMs and, in *E. coli*, a large periplasmic domain situated between TMs 1 and 2 (Kumazaki et al., 2014b). The structure of YidC is mostly solved apart from TM1, which is thought to be flexible (Figure 4). A central hydrophilic cavity which is surrounded by TMs 2–6 extends about halfway across the width of the membrane, and opens towards the cytoplasm. YidC is thought to promote TM insertion by facilitating translocation of charged loops, which bind in the hydrophilic groove. After passing a gap between TMs 2 and 4, loops are only required to traverse about half the width of the lipid bilayer (Kumazaki et al., 2014a; Wickles et al., 2014). The insertase function of YidC has been monitored by single-molecule AFM experiments, where insertion of an unfolded membrane protein attached to the AFM stylus was monitored in lipid bilayers containing YidC (Serdiuk et al., 2016; Serdiuk et al., 2017; Serdiuk et al., 2019; Laskowski et al., 2021). In these experiments, YidC alone was sufficient to enable insertion and folding of LacY, although structural elements were inserted in random order (Serdiuk et al., 2019).

In the cell, YidC is present in excess relative to SecYEG and can function as part of the holo-translocon containing YidC, SecYEG and SecDF (Schulze et al., 2014), in complex with SecYEG (Scotti et al., 2000; Nouwen and Driessen, 2002; Sachelaru et al., 2013; Sachelaru et al., 2017) or as a stand-alone insertase (Luirink et al., 2001; Samuelson et al., 2001; Serek et al., 2004; Welte et al., 2012) (Figure 4). RNCs are targeted to YidC with the help of SRP, and YidC was reported to bind to FtsY as well as to the exit tunnel of the ribosome (Driessen, 1994; Urbanus et al., 2002; Welte et al., 2012; Li et al., 2014; Geng et al., 2015; Tanaka et al., 2018). Thus, there seems to be a potential for cotranslational membrane protein targeting and insertion by YidC without the need for SecYEG in bacteria, but the details of the pathway remain poorly understood. The presence of the YidC homologue Oxa1 in mitochondria, which lack SecY, provides an obvious precedence for such a model.

The known substrates which require YidC for insertion are listed in Table 1. It is not clear, however, which of these substrates utilize YidC-only, SecYEG–YidC, or holotranslocon pathways, or whether there is any crosstalk between pathways. Only a few substrates have been identified that utilise the YidC-only pathway for membrane insertion (Table 1 and (Samuelson et al., 2000; Chen et al., 2003)). The precise role of YidC is unclear, but it has been shown to be required for the correct folding of LacY (Nagamori et al., 2004; Serdiuk et al., 2017) and stability and complex formation for MalF (Wagner et al., 2008). Since most YidC substrates are components of membrane protein complexes, YidC may act as a chaperone during complex formation in the phospholipid bilayer. So far, only the substrate MalF is known to require YidC for complex formation, so this needs to be explored for the other YidC substrates as well.

**TABLE 1:** YidC substrates and their attributes.

Protein	Topology	No. of TMs	Periplasmic domain^a^	Part of complex	SRP dependent	YidC dependent	SecYEG dependent	Reference
F_0_A	N-out	5	none	+	+	+	+	Kol et al. (2009)
F_0_B	N-out	1	none	+	+	+	+	Yi et al. (2004)
F_0_C	N-out	2	none	+	−	+	−	van Bloois et al. (2004); Yi et al. (2004); Komar et al. (2016)
NuoK	N-out	3	none	+	+	+	+	Price and Driessen, (2010)
CyoA	N-in	3	112–315	+	+	+	+	du Plessis et al. (2006)
MscL	N-in	2	46–74	−	+	+	+^b^	Facey et al. (2007); Komar et al. (2016)
TatC	N-in	6	45–75	+	+	+^c^	+^c^	Welte et al. (2012); Zhu et al. (2012)
MtlA	N-in	6	none	+	+	+^d^	+^d^	Welte et al. (2012); Deutscher et al. (2006)
FtsQ	N-in	1	49–276	+	+	+/−	+	Scotti et al. (2000); van der Laan et al. (2004); van der Laan et al. (2001)
LepB	N-out	2	78–324	−	+	+	+	Houben et al. (2004)
LacY	N-in	12	none	−	+	folding	+	Serdiuk et al. (2017)
MalF	N-in	8	93–275, 337–369, 453–483	+	+	assembly	+	Wagner et al. (2008)

+, dependent; −, independent.

a Only stretches longer than 29 amino acids are considered as a periplasmic domain.

b Efficiency of insertion is slightly higher with holotranslocon.

c Either YidC or SecYEG is sufficient for insertion, SecYEG is less efficient.

d Either YidC or SecYEG is sufficient for insertion, YidC is less efficient.

When bound to SecYEG, YidC contacts SecY at TMs 1, 2b, 3, 5 and 7, including residues inside the pore and at the lateral gate, through interactions with TM1 and loop P1 of YidC (Sachelaru et al., 2013; Petriman et al., 2018; Jauss et al., 2019). The interaction between TM1 of YidC and the SecY pore is displaced by insertion of a substrate TM, suggesting that YidC recognizes nascent protein substrates within the pore of the translocon (Sachelaru et al., 2013). Single-molecule studies of SecYEG-YidC complexes using FRET and electrophysiology measurements indicate that the complex containing YidC is more closed than SecYEG alone, although rapid fluctuations at the lateral gate are still observed by smFRET, even when YidC is present (Sachelaru et al., 2017; Mercier et al., 2021). During insertion of a membrane protein, however, YidC strongly stabilizes the open conformation of the lateral gate, although it is unclear whether YidC holds the lateral gate open, or interacts with the substrate TM in the open lateral gate (Mercier et al., 2021). In single-molecule AFM studies, insertion and folding of LacY in membranes containing SecYEG and YidC proceeds similarly to membranes containing SecYEG only, suggesting that the role of the translocon dominates over that of YidC (Serdiuk et al., 2019). The current model of YidC-dependent insertion is that TM1 of YidC occupies the channel in SecY, and is displaced by TM insertion. YidC then interacts with the lateral gate of SecY and fetches the nascent TM before it is released into the phospholipid bilayer. Any potential effects of YidC on the kinetics or specificity of membrane protein targeting await further investigations.

## Concluding Remarks

The predominant theme emerging from mechanistic studies of the cotranslational biosynthesis of membrane proteins has been that individual steps are inherently fast, and rate-limited by translation on the ribosome. Rapid targeting of RNCs to the membrane *via* the SRP pathway permits bypassing of N-terminal processing and ensures that ribosomes reach the membrane while nascent chains are still short. Subsequent insertion of TMs into the translocon and then membrane are also rapid, occurring when the nascent chain is sufficiently long that TMs can enter. This theme reinforces the concept of vectorial folding, as TMs become available to fold in the lipid bilayer in the order in which they are synthesized on the ribosome. Despite the recent progress in understanding the mechanism of membrane protein biogenesis, there are still many outstanding questions.

For instance, a clear understanding of how cytosolic loops are handled at the ribosome-translocon complex is lacking. On the one hand, there may be enough space with or without minor rearrangement to permit cytosolic loops to exit the ribosome and enter the cytosol directly. Exit of these loops may, however, cause the ribosome to leave its position on the translocon and remain tethered to the membrane by nascent protein. Such complexes may require SRP for retargeting and while there is evidence for retargeting based on selective ribosome profiling (Schibich et al., 2016), this significantly complicates the picture of membrane protein biogenesis.

During the process of TM insertion, there is evidence from several groups to suggest that entry of some TMs is delayed, and this seems to involve positively charged amino acids (Mercier et al., 2020; Nicolaus et al., 2021). Delayed entry may permit TMs to enter the lipid bilayer in pairs, rather than one at a time but it is unclear whether this is critical for efficient folding. This is difficult to disentangle, however, since positive charges are important for maintaining a correct topology and likely slow down protein synthesis.

In addition, a great deal of work has characterized ribosome pausing during mRNA translation which influences protein folding in general (reviewed in (Samatova et al., 2020)). Translational pausing can dominate the speed at which TMs enter the membrane, and could conceivably be programmed in mRNA to permit folding of complex structures before additional TMs arrive. On the other hand, if additional TMs do not interfere with initial folding steps, then pauses may simply result from the lack of any pressure to optimize translation speed at particular locations in the mRNA. Here we need a better handle on what causes translational pauses, and how likely kinetic traps are to hinder membrane protein folding.

Finally, an important outstanding question relates to which folding events and rearrangements occur inside the phospholipid bilayer. It is possible that partially folded membrane proteins remain dynamic in the lipid bilayer to permit reorganization during continued synthesis until all TMs are present. Another interesting question is at what stage membrane protein complexes start to form. Additional real-time measurements within phospholipid bilayers will be necessary to address these questions.
